# Calcium Signaling Alterations Caused by Epigenetic Mechanisms in Pancreatic Cancer: From Early Markers to Prognostic Impact

**DOI:** 10.3390/cancers12071735

**Published:** 2020-06-30

**Authors:** Cleandra Gregório, Sheila Coelho Soares-Lima, Bárbara Alemar, Mariana Recamonde-Mendoza, Diego Camuzi, Paulo Thiago de Souza-Santos, Raquel Rivero, Simone Machado, Alessandro Osvaldt, Patricia Ashton-Prolla, Luis Felipe Ribeiro Pinto

**Affiliations:** 1 Laboratório de Medicina Genômica, Centro de Pesquisa Experimental, Hospital de Clínicas de Porto Alegre, Porto Alegre 90035-007, Brazil; cleandra.gregorio@gmail.com (C.G.); barbara.alemar@gmail.com (B.A.); pprolla@gmail.com (P.A.-P.); 2 Programa de Pós-graduação em Genética e Biologia Molecular, Universidade Federal do Rio Grande do Sul, Porto Alegre 91501-970, Brazil; 3 Programa de Carcinogênese Molecular, Instituto Nacional de Câncer, Rio de Janeiro 20231-050, Brazil; sheilacoelho@gmail.com (S.C.S.-L.); drdcamuzi@gmail.com (D.C.); 4 Instituto de Informática, Universidade Federal do Rio Grande do Sul, Porto Alegre 91501-970, Brazil; mrmendoza@inf.ufrgs.br; 5 Núcleo de Bioinformática, Hospital de Clínicas de Porto Alegre, Porto Alegre 90035-007, Brazil; 6 Laboratório de Hanseníase, Instituto Oswaldo Cruz, Fiocruz, Rio de Janeiro 21040-360, Brazil; pthiagoss@gmail.com; 7 Serviço de Patologia, Hospital de Clínicas de Porto Alegre, Porto Alegre 90035-007, Brazil; rrivero@hcpa.edu.br (R.R.); smsmachado@hcpa.edu.br (S.M.); 8 Departamento de Patologia, Faculdade de Medicina, Universidade Federal do Rio Grande do Sul, Porto Alegre 90035-003, Brazil; 9 Grupo de Vias Biliares e Pâncreas, Cirurgia do Aparelho Digestivo, Hospital de Clínicas de Porto Alegre, Porto Alegre 90035-007, Brazil; aosvaldt@hcpa.edu.br; 10 Programa de Pós-graduação em Medicina: Ciências Cirúrgicas, Universidade Federal do Rio Grande do Sul, Porto Alegre 90035-007, Brazil; 11 Faculdade de Medicina, Universidade Federal do Rio Grande do Sul, Porto Alegre 90035-007, Brazil; 12 Departamento de Bioquimica, Instituto de Biologia Roberto Alcantara Gomes, Universidade do Estado do Rio de Janeiro, Rio de Janeiro 20550-900, Brazil

**Keywords:** pancreatic ductal adenocarcinoma, calcium signaling pathway, DNA methylation, early methylation alterations, prognostic biomarkers

## Abstract

Pancreatic ductal adenocarcinoma (PDAC) is an aggressive disease with high mortality rates. PDAC initiation and progression are promoted by genetic and epigenetic dysregulation. Here, we aimed to characterize the PDAC DNA methylome in search of novel altered pathways associated with tumor development. We examined the genome-wide DNA methylation profile of PDAC in an exploratory cohort including the comparative analyses of tumoral and non-tumoral pancreatic tissues (PT). Pathway enrichment analysis was used to choose differentially methylated (DM) CpGs with potential biological relevance. Additional samples were used in a validation cohort. DNA methylation impact on gene expression and its association with overall survival (OS) was investigated from PDAC TCGA (The Cancer Genome Atlas) data. Pathway analysis revealed DM genes in the calcium signaling pathway that is linked to the key pathways in pancreatic carcinogenesis. DNA methylation was frequently correlated with expression, and a subgroup of calcium signaling genes was associated with OS, reinforcing its probable phenotypic effect. Cluster analysis of PT samples revealed that some of the methylation alterations observed in the Calcium signaling pathway seemed to occur early in the carcinogenesis process, a finding that may open new insights about PDAC tumor biology.

## 1. Introduction

Pancreatic cancer is a very aggressive disease, with 5-year survival rates below 8% and a strong ability to metastasize even before the primary tumor is clinically detected [1,2]. Tumor aggressiveness, lack of specific symptoms in early stages, and resistance to cytotoxic drugs, all contribute to the high mortality rates [3]. In fact, current estimates predict that pancreatic cancer will be the second most lethal tumor by 2030 [4]. Considering all pancreatic malignancies, pancreatic ductal adenocarcinoma (PDAC) is the most prevalent type, accounting for more than 90% of the cases [5]. PDAC initiation and progression are promoted by the interaction between driver mutations and the disruption of epigenetic regulatory circuits, such as DNA methylation [6].

DNA methylation is one of the best understood epigenetic mechanisms of transcriptional regulation. In cancer, the DNA methylation landscape often involves global hypomethylation mainly described at CpG sites located in intergenic regions, including repetitive elements [7]. Alternatively, studies using RefSeq gene analysis (such as the 450 K BeadChip Array platform) show that most CpGs are hypermethylated in cancer, affecting mainly tumor suppressor genes [8,9]. However, there are only limited data on the wide DNA methylation patterns of PDAC [10,11].

The discovery of new biomarkers including DNA methylation and other biologic processes for the development of novel target-driven therapies, and the definition of prognosis in PDAC are urgent needs. In this study, we aimed to characterize the PDAC DNA methylome in search of novel altered pathways associated with tumor development through a comparative analysis of tumor and non-tumor pancreatic tissue (PT) samples. An important new finding resulting from this analysis was the identification of several differentially methylated genes of the calcium (Ca^2+^) signaling pathway, linked to key pathways in pancreatic carcinogenesis. In addition, some of the methylation alterations observed in this pathway seem to occur early in the carcinogenesis process, a finding that may open new insights about PDAC tumor biology.

## 2. Results

### 2.1. Genome-Wide DNA Methylation Profile in Pancreatic Adenocarcinoma

**Figure 1:**
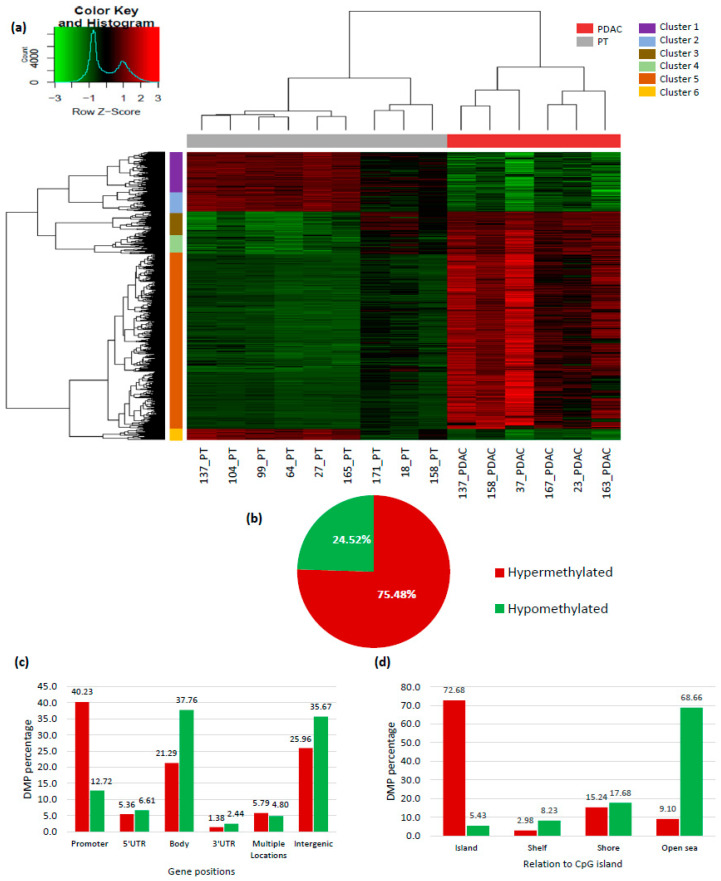
DNA methylation profile of pancreatic ductal adenocarcinoma. (**a**) Heatmap showing the unsupervised hierarchical clustering of pancreatic ductal adenocarcinoma (PDAC, with a minimum 70% cellularity) and non-tumoral adjacent pancreatic tissue (PT) according to the methylation profile of the 10,361 probes found to be differentially methylated between groups (adjusted *p*-value < 0.01 and ∆β ≥ 0.2). Hyper- and hypomethylation are represented in red and green, respectively. Colored bars on the left side of the heatmap represent probe clusters defined by their methylation similarities; (**b**) pie chart showing the percentage of hypomethylated and hypermethylated probes in PDAC relative to PT; (**c**) overall distribution of hypo- and hypermethylated probes according to their gene position; (**d**) overall distribution of hypo- and hypermethylated probes according to their relation to CpG islands.

The genome-wide DNA methylation profile of six PDACs (with a minimum of 70% cellularity) compared to nine PT fresh frozen tissue samples was determined in an exploratory cohort using Infinium 450 K beadchips. The unsupervised hierarchical clustering analysis of the 10,361 differentially methylated probes (DMPs, adjusted *p*-value < 0.01 and ∆β ≥ 0.2) exhibited clear separation between the PDAC and PT. As shown in Figure 1a, two major clusters emerged: the first comprised PT samples and was predominantly hypomethylated, while the PDAC samples formed the second cluster that showed an overall hypermethylated profile (75.48% of the DMPs). PDAC hypermethylated probes were most frequently annotated to promoter regions (40.23%), while most hypomethylated probes were mapped to gene bodies (37.76%). Stratification by distance to CpG islands revealed that hypomethylated CpG sites most commonly encompassed open sea regions (68.66%), and hypermethylation was most common at CpG islands (72.68%) (Figure 1b–d).

### 2.2. Ca^2+^ Pathway Genes Are Epigenetically Altered in PDAC

**Figure 2:**
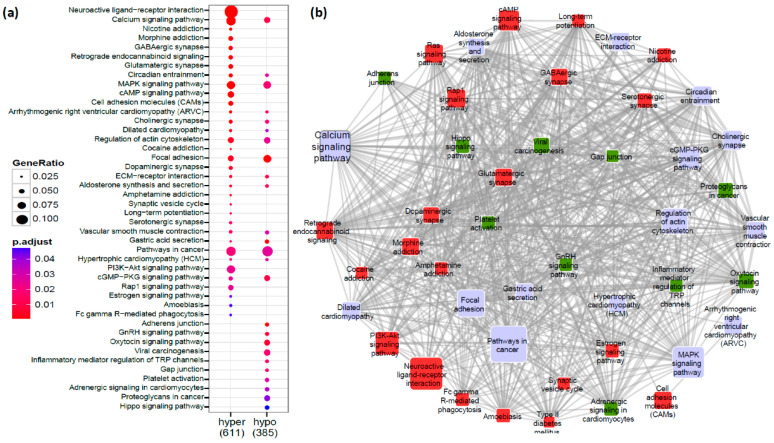
Pathway enrichment analysis of differentially methylated genes (*n* = 996) in pancreatic ductal adenocarcinoma. (**a**) Functional enrichment of the hypermethylated (*n* = 611) and hypomethylated genes (*n* = 385) annotated from the differentially methylated probes (DMPs); (**b**) interactions between the enriched pathways evidencing the number of shared differentially methylated genes. The pathways in red and green are those enriched for hyper- and hypomethylated genes, respectively, and those in lilac are enriched for both methylation profiles.

The next step was to analyze the potential biological relevance of the differentially methylated CpG sites identified in the supervised comparison between PDAC and PT samples. The DMPs mapped to 2715 genes, 1766 hypermethylated and 1100 hypomethylated, with an overlap of 151 genes among both sets. The set of 2715 genes had 611 hypermethylated and 386 hypomethylated genes annotated in the Kyoto Encyclopedia of Genes and Genomes (KEGG) database that were used as query gene sets to assess the functional enrichment of DMPs. Hypermethylated and hypomethylated genes were significantly associated with the enrichment of 36 and 25 cellular pathways, respectively (Figure S1a,b, Tables S1 and S2). Subsequently, we merged both analyses to identify the biological pathways that were frequently deregulated in PDAC by both hyper- and hypomethylation. As shown in Figure 2a, several pathways well known to be involved in cancer development were identified, such as the MAPK signaling pathway and the focal adhesion pathway [12,13]. Additionally, we observed that the Ca^2+^ signaling pathway had a large number of genes significantly hypo- and hypermethylated. This pathway is intrinsic to multiple aspects of cancer biology, such as tumor initiation, metastasis, and drug resistance [14]. The overlap between the differentially methylated genes of the Ca^2+^ signaling and other cellular pathways was investigated, and revealed that several of them were shared with other significantly enriched pathways, such as the Hippo and Ras signaling pathways (Figure 2b).

### 2.3. Validation Cohort

**Figure 3:**
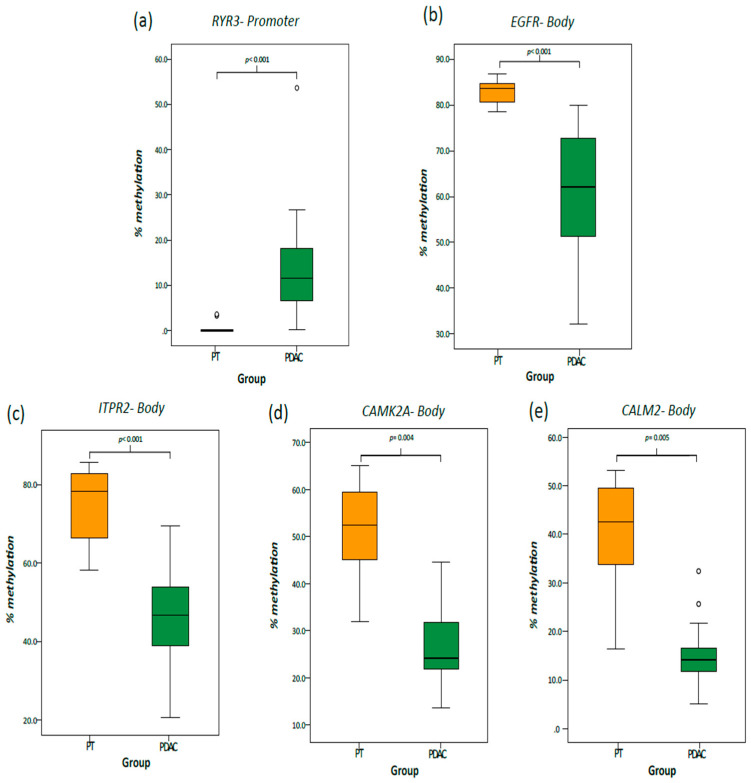
Validation of the genome-wide DNA methylation results by pyrosequencing. Boxplots represent the overall methylation of pancreatic adenocarcinoma (PDAC, *n* = 43) and non-tumoral adjacent pancreatic tissue (PT, *n* = 24) samples for the following genes: (**a**) *RYR3* (promoter); (**b**) *EGFR* (gene body); (**c**) *ITPR2* (gene body), (**d**) *CAMK2A* (gene body); and (**e**) *CALM2* (gene body).

The methylation profile of five genes were assessed in a validation cohort made of 43 PDAC and 24 PT samples, with the clinical features of patients shown in Table S3. The five genes were selected with the following criteria: those with a previously described role in cancer and containing DMPs associated with a high |∆β|. One hypermethylated promoter (*RYR3*, Ryanodine Receptor 3) and four hypomethylated gene body probes (*EGFR*, Epidermal Growth Factor Receptor; *ITPR2*, Inositol 1,4,5-Trisphosphate Receptor Type 2; *CAMK2A*, Calcium/Calmodulin Dependent Protein Kinase II Alpha; and *CALM2*, Calmodulin 2) were chosen. Methylation levels at the CpG sites interrogated by the Infinium probe as well as surrounding CpG sites were evaluated, and the β-values obtained by pyrosequencing were strongly correlated with the Infinium assay (Figure 3 and Figure S2).

### 2.4. Correlation between Methylation and Expression of Ca^2+^ Pathway Genes

**Figure 4:**
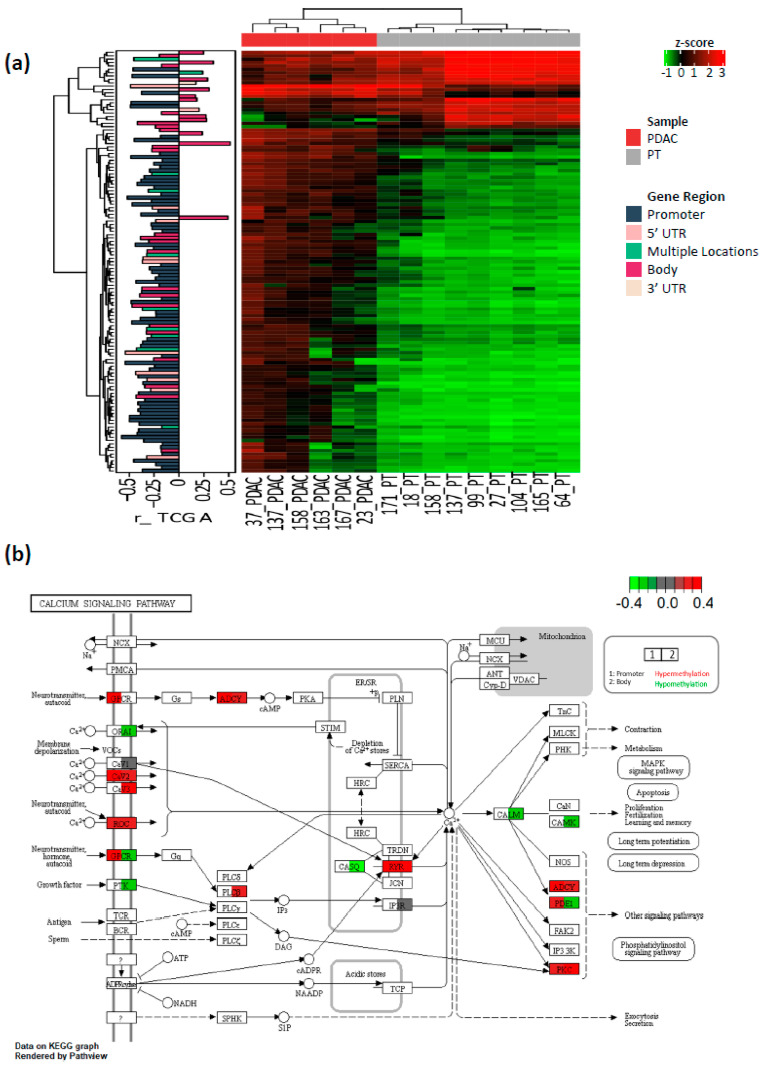
Calcium (Ca^2+^) signaling pathway analysis. (**a**) Heatmap showing the methylation profile of the probes (*n* = 112 probes) annotated to genes involved in the Ca^2+^ signaling pathway for which a significant correlation (false discovery rate (FDR)-adjusted *p-*value < 0.05) with a gene expression was observed in the TCGA dataset (The Cancer Genome Atlas, *n* = 141). Heatmap was built based on the methylation data generated in the present study. The bars located on the left side of the heatmap indicate the correlation coefficient per gene region affected by differential methylation, and is based on the TCGA data; (**b**) schematic diagram of the Ca^2+^ signaling pathway. The network was built based on the Kyoto Encyclopedia of Genes and Genomes (KEGG) pathway map (KEGG: hsa04020). Each box represents a group of gene products (mostly protein) that have a common function, and its nomenclature can include one or many gene products. Gene products regarding each box are represented in Table S4. Ca^2+^ signaling pathway genes for which a significant correlation between methylation and expression was observed are highlighted showing the differential methylation in promoters and gene bodies identified in the present study. The red squares indicate hypermethylated genes while the green squares represent hypomethylated genes. UTR: Untranslated Region.

The methylation profile of five genes were assessed in a validation cohort made of 43 PDAC and 24 PT samples, with the clinical features of patients shown in Table S3. The information available in TCGA (The Cancer Genome Atlas) about PDAC was used to explore the impact of DNA methylation on the expression of the Ca^2+^ signaling pathway genes [15]. Among 173 DMPs in the Ca^2+^ pathway genes, 112 (64.73%) showed a significant correlation with gene expression (Table S4). The bars located on the left side of the heatmap (Figure 4a) indicate the correlation coefficient per gene region affected. The methylation levels of probes annotated to promoters were significantly and inversely correlated with the expression in 62.3% of the comparisons. Comparatively, the methylation profile of probes located in gene bodies showed a correlation with an expression of 59.6% in the comparisons, 64.3% inverse and 35.7% positive.

In Figure 4b, a schematic diagram of the Ca^2+^ signaling pathway based on the KEGG database shows groups of genes (grouped within boxes by function, as indicated in Table S4) with significant correlations between methylation and expression. Colored boxes indicate the differential methylation in the promoters and gene bodies identified in the present study. Genes that control intracellular Ca^2+^ storage, such as ryanodine receptors (*RYR2*, Ryanodine Receptor 2, and *RYR3*), were hypermethylated (promoters and gene bodies), while the inositol 1,4,5- trisphosphate receptor (*ITPR1*, Inositol 1,4,5-Trisphosphate Receptor Type 1) showed a heterogeneous DNA methylation profile in the body region.

### 2.5. Differential Expression of Ca^2+^ Pathway Genes Is Potentially Associated with Survival

Ca^2+^ pathway genes that displayed significant correlations between methylation and expression in TCGA pancreatic cancer samples (Table S4) were selected for survival analysis. We evaluated the expression impact of 40 genes on a PDAC patient’s overall survival (OS) (Table S5), including two genes that had already been investigated in the validation cohort (*CALM2*, and *RYR3*).

In the TCGA dataset (*n* = 146), pathologic N stage (N1 vs. N0, Hazard Ratio (HR) = 1.832 (1.039–3.23 95%CI), *p* = 0.0364) and residual tumor status after surgical resection (R1 vs. R0, HR = 1.899 (1.152–3.131 95%CI), *p* = 0.012) were associated with OS, and remained in the model.

In the univariate analysis, PDAC patients with high *ADRA1A* (Adrenoceptor Alpha 1A), *CACNA1A* (Calcium Voltage-Gated Channel Subunit Alpha1 A), *CACNA1B* (Calcium Voltage-Gated Channel Subunit Alpha1 B), *CACNA1H* (Calcium Voltage-Gated Channel Subunit Alpha1 H), *CASQ2* (Calsequestrin 2) *ORAI2* (ORAI Calcium Release-Activated Calcium Modulator 2), *P2RX2* (Purinergic Receptor P2X 2), *PDE1C* (Phosphodiesterase 1C), and *PRKCB* (Protein Kinase C Beta) expression showed increased OS when compared to those with a low expression (Table S5). Conversely, decreased *HRH1* (Histamine Receptor H1) expression was associated with increased OS (Table S5).

**Figure 5:**
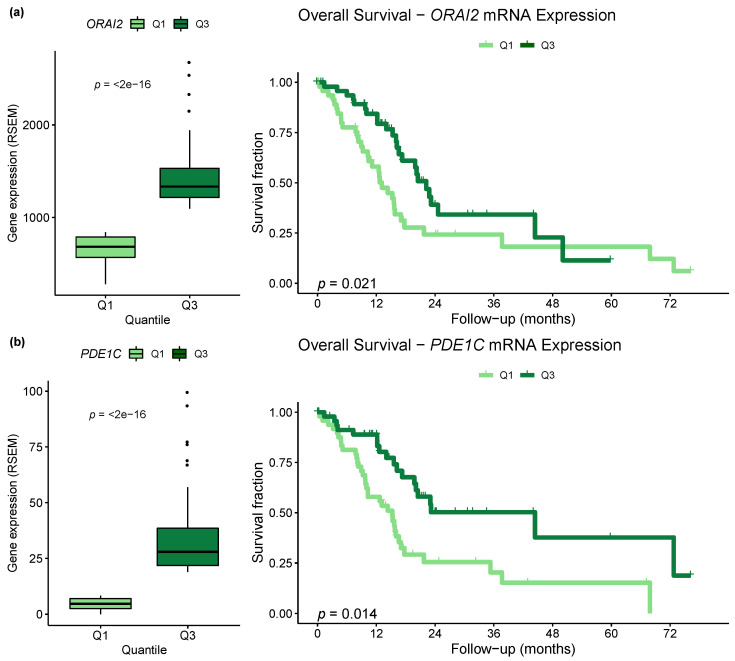
Impact of the expression of Ca^2+^ signaling genes on PDAC overall survival. mRNA expression of selected genes was divided by tertiles, and the lower (Q1) and higher (Q3) tertiles were used to classify the cases into low and high expression, respectively. The adjustments were performed for pathologic N stage and residual tumor status after surgical resection. (**a**) On the left side of the Figure, the boxplot representing the expression profile of *ORAI2* in the low (Q1) and high (Q3) expression groups of PDAC patients. On the right side, the Kaplan–Meier survival curve shows the differences between the groups. Hazard Ratio (HR) = 0.5004 (0.278–0.9009 95%CI), *p* = 0.021. (**b**) On the left side of the Figure, the boxplot representing the expression profile of *PDE1C* in the low (Q1) and high (Q3) expression groups of PDAC patients. On the right side, the Kaplan–Meier survival curve shows the differences between the groups. HR = 0.4625 (0.2493–0.8577 95%CI), *p* = 0.0144. RSEM, RNA-Seq by Expectation-Maximization.

After COX regression, the *ORAI2* (adjusted HR = 0.5004 (0.278–0.9009 95%CI), adjusted *p* = 0.021) and *PDE1C* (adjusted HR = 0.4625 (0.2493–0.8577 95%CI), adjusted *p* = 0.0144) higher expressions were shown to be independent variables associated with a better OS (Figure 5, Table S5).

### 2.6. Aberrant Methylation in Ca^2+^ Genes Is an Early Change in Pancreatic Cancer

We investigated the overall methylation profile of three PT samples (18 PT, 158 PT, and 171 PT) with intermediate methylation levels at the DMPs between PDAC and PT (Figure 1a, these samples will be named PT-like from now on). To validate this discordant methylation profile, first a multidimensional scaling plot was used, showing that PT-like samples were more closely related to PDAC than PT samples, when considering principal component 1 (Figure S3a). This finding may be associated with molecular field cancerization, since these samples showed a high percentage of normal ducts (>80%), small focal fibrosis regions, and no evidence of neoplastic cell contamination (Figure S3b–d).

**Figure 6:**
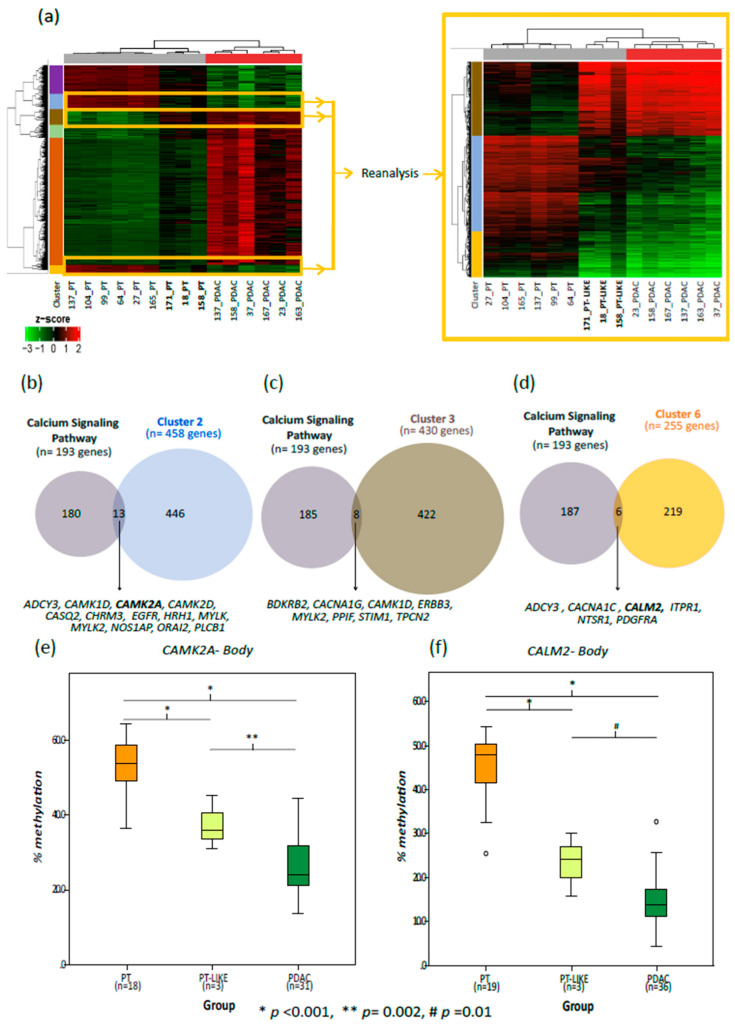
Comparative DNA methylation analysis of the PT, PT-like and the PDAC samples. (**a**–**d**) Tissue samples with ≥70% cellularity. (**a**) Heatmaps highlighting the methylation profile of the probe clusters supposed to be altered in early stages of PDAC development. Heatmap on the left shows the 10,361 DMPs between the PDAC and PT. Clusters 2, 3 and 6 were selected for a detailed analysis (heatmap on the right) since their probes showed intermediate methylation levels (between PDAC and PT) in the PT-like samples; (**b**–**d**) the overlap between the genes involved in the calcium signaling pathway and the genes belonging to each selected probe cluster ((**b**) cluster 2; (**c**) cluster 3; and (**d**) cluster 6). (**e**,**f**) The boxplots representing the overall methylation level of *CALM2* (**e**) and *CAMK2A* (**f**) in the PT, PT-like and the PDAC samples (≥20% cellularity). *p*-values were calculated using the generalized estimating equations.

Field cancerization is defined by a set of genetic and epigenetic alterations that indicate that a specific tissue area is undergoing a transformation process, or has a predisposition to initiate such a process, and this may occur without overt morphological changes [16]. Considering that this process occurred in PT-like samples, we performed a deep analysis of DMP clusters, comparing the major similarities between PDAC and PT-like. Three clusters derived from the previous unsupervised hierarchical clustering analysis were selected for further investigation, namely, clusters 2, 3, and 6. After a new unsupervised hierarchical clustering analysis, using only the DMPs of these clusters, the PT-like samples were grouped with PDAC samples (Figure 6a). Then, we performed the pathway enrichment analysis with the corresponding annotated genes and observed that 24 differentially methylated genes were involved in the Ca^2+^ signaling pathway: thirteen genes from cluster 2, eight from cluster 3, and six from cluster 6; and three genes appeared in more than one cluster (Figure 6b–d, Table S6). The stepwise methylation profile in the PT, PT-like and the PDAC samples was observed in two previously validated genes (*CALM2* and *CAMK2A*, Figure 6e,f) as well as in other 22 genes from the Ca^2+^ signaling pathway (Figure S4).

## 3. Discussion

In this study, we used a genome-wide DNA methylation approach to identify new pathways associated with PDAC development. Pathway enrichment analysis revealed the differentially methylated genes of the Ca^2+^ signaling pathway. Moreover, we showed aberrant DNA methylation patterns in a few PT samples indicating the possible formation of field cancerization. Nones et al. and Mishra et al. previously used the 450 K BeadChip Array to build two PDAC methylome databases, and similar to our data (Figure 1b), they showed that the majority of DMPs in PDAC were hypermethylated (56.59% and 50.59%, respectively) and located in promoter regions [10,11]. CpG island methylation in promoters is frequently associated with gene silencing during tumorigenesis, providing an alternative mechanism to mutations by which tumor suppressor genes may be inactivated within a cancer cell [17,18]. In our exploratory cohort, as well as in other studies, the Tumor Suppressor Genes (TSGs) *NPTX2* (Neuronal Pentraxin 2), *CDO1* (Cysteine Dioxygenase Type 1), *TFPI2* (Tissue Factor Pathway Inhibitor 2), *SFRP1* (Secreted Frizzled Related Protein 1), *SFRP2* (Secreted Frizzled Related Protein 2), *PENK* (Proenkephalin) and *FOXE1* (Forkhead box E1) had a remarkable hypermethylation pattern in the PDAC group [19,20,21,22,23,24].

Key signaling pathways involved in cancer were identified as differentially methylated in our study, e.g., the MAPK signaling pathway, which is engaged in multiple proliferative cellular processes (cell differentiation, proliferation, and apoptosis) [12], and the focal adhesion pathway, which may play a role in the development and progression of cancer [13]. In addition, we observed that the Ca^2+^ signaling pathway had a high number of genes both significantly hypo- and hypermethylated (Figure 2a). Ca^2+^ is a ubiquitous intracellular messenger that controls diverse processes in cellular physiology, such as gene transcription, cell progression, cell motility, and apoptosis [25]. Although Ca^2+^ pathway methylation in PDAC is still poorly explored, many of its genes have already been described as differentially methylated in other solid tumors, including gastric [8], prostate [8,26], and breast cancer [8,26]. Resting cytosolic free Ca^2+^ is maintained at lower levels than those of extracellular space, and its equilibrium dynamics is carefully regulated by the plasmatic membrane, endoplasmic reticulum (ER), and mitochondria [27], using a “toolkit” of channels, pumps, and cytosolic buffers to control Ca^2+^ cell homeostasis [28]. The spatial and temporal dynamics of Ca^2+^ signaling results in specific cellular responses mediated by the activation of a subset of Ca^2+^-dependent effectors [29].

During carcinogenesis, several cellular metabolic functions become deregulated, including those related to Ca^2+^ signaling [30,31], and therefore, it is not surprising that changes in the expression or function of Ca^2+^ handling proteins impact tumorigenesis. In fact, different tumors present altered expression or mutations in the genes involved in Ca^2+^ signaling [32,33,34,35,36]. Different Ca^2+^ channels or pumps are potential therapeutic targets in different cancer subtypes, and are correlated with prognosis [8,32,37,38]. In our study, we showed that altered methylation levels of *ADRA1A*, *CACNA1A*, *CACNA1B*, *CACNA1H*, *CASQ2*, *HRH1*, *ORAI2*, *P2RX2*, *PDE1C*, and *PRKCB*, had an impact on their expression (Table S4). Furthermore, *PDE1C* promoter hypermethylation (Table S4, positive ∆beta, and negative r correlation) and *ORAI2* gene body hypomethylation (Table S4, negative ∆beta, and negative r correlation) resulted in their downregulated expression in PDAC samples, identified as independent predictors of lower overall survival (Figure 5 and Table S5). Although we did not perform protein expression analyses (due to the limitation of the tissue samples available), the correlation between DNA methylation and mRNA expression, and the consequent impact on prognosis, which suggests a phenotypic importance of this finding. Interestingly, ORAI2 controls Ca^2+^ influx through the plasmatic membrane [32,39,40].

Scarce information is available regarding the methylation-related expression deregulation of genes involved in the Ca^2+^ signaling pathway in PDAC. In one of the few studies, the methylation profile of *S100A4* (S100 Calcium Binding Protein A4), a Ca^2+^-binding protein previously implicated in metastasis [41], was evaluated in PDAC samples and cell lines. Hypomethylation was detected in tumors, whereas all normal pancreatic tissue samples analyzed were hypermethylated in the same region. Moreover, gene and protein expression patterns were correlated with the methylation profile, and were associated with poor tumor differentiation [42]. In addition, methylation patterns of *PCDH10* (Protocadherin 10), a member of the non-clustered protocadherin family that plays an important role in Ca^2+^-dependent cell–cell signal transduction and adhesion, was investigated in PDAC cell lines (Capan-1, Panc-1, AsPC-1 and BxPC-3). *PCDH10* promoter methylation was observed in 50% of the cell lines studied and resulted in a marked reduction of its expression [43]. Later, this tumor suppressor gene was investigated in other PDAC cell lines and, as expected, the *PCDH10* promoter methylation again correlated with reduced protein expression. This study also showed high methylation levels in clinical samples (*n* = 23), and the presence of this methylation pattern was associated with reduced progression-free survival [44]. Using the TCGA data, Mishra et al. analyzed a genome-wide DNA methylation profile, and although the genes of the Ca^2+^ signaling pathway occupied the fourth position in the functional enrichment of differentially methylated probes, this finding was not further explored [11]. In addition, also using the TCGA data, another study combined the methylation and expression data of twelve solid tumors (no PDAC included) in order to identify common patterns of methylation. Alterations in the Ca^2+^ signaling pathway were observed in nine cancer types and *AGTR1 GRIN2A* (Glutamate Ionotropic Receptor NMDA Type Subunit 2A), *ITPKB* (Inositol-Trisphosphate 3-Kinase B), and *SLC8A3* (Solute Carrier Family 8 Member A3) were repressed by hypermethylation in six of them [8].

Cytoplasmic Ca^2+^ concentrations rise in response to a variety of stimuli that activate Ca^2+^ channels in the plasma membrane (as ORAI, CaV1,2 and 3), or by release from intracellular stores through stromal interaction molecule (STIM), inositol 1,4,5-trisphosphate receptors (IP_3_R), and ryanodine receptors (RYRs). *ORAI2*, *CACNA1C (*Calcium Voltage-Gated Channel Subunit Alpha1 C*)*, *CACNA1G* (Calcium Voltage-Gated Channel Subunit Alpha1 D), *STIM1* (Stromal interaction molecule 1) and *ITPR1* (Inositol 1,4,5-Trisphosphate Receptor Type 1) had an altered methylation profile in the PT-like samples (Figure 6b–d and Figure S4). ORAI1 and STIM1 are known as calcium release-activated calcium (CRAC) channels [45]. In PDAC cell lines, they are overexpressed and play a pro-survival anti-apoptotic role in this tumor [46]. In a recent study, Khan et al. have demonstrated that PDAC proliferation suppression is possible by targeting the CRAC channel with RP4010, a CRAC channel inhibitor [47]. Growth factors binding to tyrosine kinase receptors (e.g., epidermal growth factor receptor, EGFR) and G protein-coupled receptors (GPCRs; e.g., histamine receptor H1, HRH1) control the intracellular Ca^2+^ by plasma membrane. After binding, phospholipase C is activated and promotes the generation of inositol-1,4,5-trisphosphate (IP_3_) and diacylglycerol, which lead to the release of Ca^2+^ from the ER into the cytosol by intracellular channels (such as ITPR1 and ITPR2) [48]. In addition to *ITPR2*, *EGFR* is frequently mutated or overexpressed in various cancer types [49,50], and in PDAC particularly, *EGFR* is essential for KRAS*-*driven pancreatic carcinogenesis [51,52,53]. It is known that *KRAS*-activating mutations are early events in PDAC carcinogenesis and occur in ~90% of cases [54]. RAS proteins can also activate phospholipase C and generate IP3, leading to Ca^2+^ influx, so the most frequently mutated gene in PDAC is directly linked to Ca^2+^ signaling.

RYRs represent another way to control Ca^2+^ ER store, and in this study, *RYR2* and *RYR3* were found hypermethylated in PDAC, and their methylation levels were inversely correlated with mRNA expression (Table S4). These receptors are regulated by Ca^2+^ voltage channels and by various ions, molecules and proteins, e.g., Ca^2+^, Mg^2+^, calmodulin (CALM), Ca^2+^/calmodulin- dependent protein kinase II (CAMK2), and nicotine [55,56]. Once Ca^2+^ levels rise in the cytoplasm, they are strictly controlled by CALM. Ca^2+^ binding dramatically changes the conformation of CALM and increases its affinity for a large number of CALM-binding proteins, including the multifunctional CALM kinases such as CAMK2 [57]. CAMK2 phosphorylates nearly 40 different proteins, including enzymes, ion channels, kinases, and transcription factors [58], and it is overexpressed in digestive cancers, such as colorectal cancer [59,60]. Epithelial–mesenchymal transition (EMT), one of the cancer hallmarks, is also controlled by Ca^2+^ levels through CAMK2A. Focal adhesion kinase (FAK), which increases the turnover of cell–cell contacts, is phosphorylated by CAMK2 and is consequently upregulated [61]. It is important to mention that the FAK pathway was differentially methylated in our analysis (Figure 2a).

On top of its association with different aspects of cancer cell biology and its crosstalk with other key signaling pathways, a role for the activation of Ca^2+^ signaling in therapy resistance has also been proposed. The mechanisms involved include the induction of multi-drug resistance (MDR) proteins via the Ca^2+^-dependent transcription factor NFAT and the acquisition of stemness phenotypes by the induction of pluripotency genes and EMT [62,63,64]. In pancreatic cancer, ORAI3 and STIM1 (key components of store operated calcium entry—SOCE) have been shown to be required for TGF-β-dependent SNAIL transcription [65] and TGF-β signaling has already been associated with stroma-mediated drug resistance [66]. The EMT induction of pancreatic cells can also be mediated by acid-sensing ion channels (ASICs). ASICs sense the extracellular acidification (common in pancreatic cancer) and in response, increase intracellular Ca^2+^ levels and activate RhoA signaling [64]. Furthermore, Na(+)/H(+) exchangers, such as NHE1 (sodium/proton exchanger 1), interact with calmodulin in a Ca^2+^-dependent manner and become activated, promoting intracellular alkalization and extracellular acidification [67]. Therefore, a complex positive feedback loop involving Ca^2+^ signaling, TGF-β, and microenvironment acidification might be involved in EMT induction and, consequently, therapy resistance in pancreatic cancer.

It is important to mention that extracellular acidification is also involved in immune escape, which has been associated with lymph node metastasis in pancreatic cancer and is being explored as one of the most promising targets for therapy in cancer [64,68,69]. Besides the previously mentioned mechanisms of microenvironment acidification, *KRAS* mutations and the consequent activation of MAPK and PI3K-mTOR signaling can lead to GLUT1 upregulation. This glucose transporter can also be induced by nutrient deprivation and hypoxia. Consequently, high rates of aerobic glycolysis result in lactate production and microenvironment acidification. This not only blocks lactate export by T-cells, which also use aerobic glycolysis as an energetic source, but also downregulates interferon γ (IFN-γ) production by T-cells and natural killer cells, and polarizes macrophages to an immunosuppressive phenotype [70]. At the same time, tumor cells can cope with high extracellular acidification by modulating the expression of pH-regulating proteins and they can also use lactate as an alternative energetic source [70]. Hypoxia and the glycolytic metabolism were also associated with a wide range of epigenetic alterations both in tumor and immune cells within the tumor microenvironment, as shown in different cancer models, and HIF-2α (hypoxia-inducible factor 2 alpha) interaction with beta-catenin was recently shown in pancreatic cancer [71,72]. Indeed, immune tolerance and lymph node invasion have also been linked to the activation of the Wnt signaling pathway in PDAC [68,73]. This pathway is one of the central players in the acquisition of stemness phenotypes and one of its non-canonical forms involves Ca^2+^ signaling [74]. Altogether, these data suggest another connection between the alterations identified in the present study, immune escape and local dissemination in PDAC.

Based on the widespread dysregulation of Ca^2+^ signaling in tumors and on its impact on different steps of tumor development and progression, Ca^2+^ signaling receptors have been suggested as therapeutic targets for different types of cancer. The blockage of TRPVs (transient receptor potential vanilloid channels), T-type Ca^2+^ channels, ORAIs and TRPCs (transient receptor potential channels) have been shown to suppress the proliferation/invasion and induce cell death in different cancer models [75]. In this context, the drug repurposing of already available blockers of SOCE and T-type Ca^2+^ channels have been evaluated [76,77]. Conversely, the activation of overexpressed targets may also be an alternative for inducing cell death, as shown in breast and prostate cancer [78,79]. Therapy targeting the Ca^2+^ signaling pathway in combination with conventional chemo or radiotherapy has also been proposed. Chemotherapy drugs approved for pancreatic cancer treatment, such as Fluorouracil (5-FU) and platins, have been tested in combination with ORAI1 (ORAI Calcium Release-Activated Calcium Modulator 1) silencing and T-type Ca^2+^ channels blockers in hepatocellular carcinoma and ovarian cancer, respectively, with promising effects [80,81]. Nevertheless, our findings suggest that in vitro studies should be carried out to understand the role of Ca^2+^ pathway altered genes in pancreatic carcinogenesis before pre-clinical assays with some of these drugs are developed.

Another relevant aspect to be considered before proposing a targeted therapy against the Ca^2+^ signaling pathway is its participation in the maintenance of different physiological systems. Therefore, although Ca^2+^ signaling represents a complex pathway involved in the establishment of different cancer phenotypes, and its therapeutic targeting seems promising, it might result in a plethora of side effects. In this context, and based on the widespread alterations of DNA methylation affecting different central signaling pathways in pancreatic cancer as shown here, epigenetic therapy may represent an alternative option. This is especially relevant because a crosstalk between Ca^2+^ signaling and DNA methylation has already been described. Ca^2+^ signaling is essential to initiating epigenetic reprogramming in early embryogenesis [82], and candidate epigenetic drugs have been shown to induce the reactivation of TSGs and cell death via a Ca^2+^/CAMK-dependent pathway [38]. In addition to TSGs reactivation, DNA methylation inhibitors have also been shown to induce the expression of cancer-testis genes and transposable elements in cancer cells, resulting in the expression of neoantigens and in a viral mimicry state [83,84,85]. These effects, together with the reprogramming of exhausted T-cells, put epigenetic therapy as a very promising approach in combination with immune checkpoint blockade for PDAC [85].

Finally, our data suggest that the dysregulation of Ca^+2^ signaling by epigenetic mechanisms is an early event in pancreatic carcinogenesis, reinforcing its relevance in this process. The stepwise methylation profile of *HRH1*, *CASQ2*, and *ORAI2*, together with twenty-one other genes that control the Ca^2+^ influx, were found in PT-like samples (Figure 4b–d and Figure S4, Table S6). However, these alterations need to be validated in an independent longitudinal study, since they may not only shed light on the early molecular mechanisms driving PDAC development, but also represent potential biomarkers. DNA methylation alterations have been proposed as useful biomarkers due to their high sensitivity and specificity, and because they can be assessed by minimally invasive approaches, such as in liquid biopsies [86]. Therefore, our results bring the epigenetic dysregulation of the Ca^+2^ signaling pathway as a promising tool for PDAC management, either as diagnostic and prognostic biomarkers, or as therapeutic targets.

## 4. Materials and Methods

### 4.1. Patients and Sample Collection

Patients enrolled in this study were treated at Hospital de Clínicas de Porto Alegre (HCPA/UFRGS), in Southern Brazil between 2012 and 2018. Inclusion criteria were: pathology-proven diagnosis of PDAC, and no history of previous or current chemo- or radiation therapy. PDAC and PT samples were obtained during surgery or diagnostic biopsy procedures, and stored according to the biobank protocols from the hospital. Hematoxylin–eosin slides were prepared for all cases to confirm the diagnosis and assess the sample quality, which was performed by two pathologists (R.R. and S.M.). Samples with pancreatitis, necrosis, fibrosis and less than 20% cellularity were excluded [10]. The study was conducted in accordance with the Declaration of Helsinki, and the protocol was approved by the Ethics Committee of Hospital de Clínicas de Porto Alegre (Project Number: 2014–0526). All subjects gave their informed consent for inclusion before they participated in the study. Information about age at diagnosis, gender, TNM Classification of Malignant Tumors (TNM), classification, tumor location, and differentiation grade were obtained from the patient’s electronic medical record. Tumor location was categorized as pancreatic head vs. non-head.

**Scheme 1:**
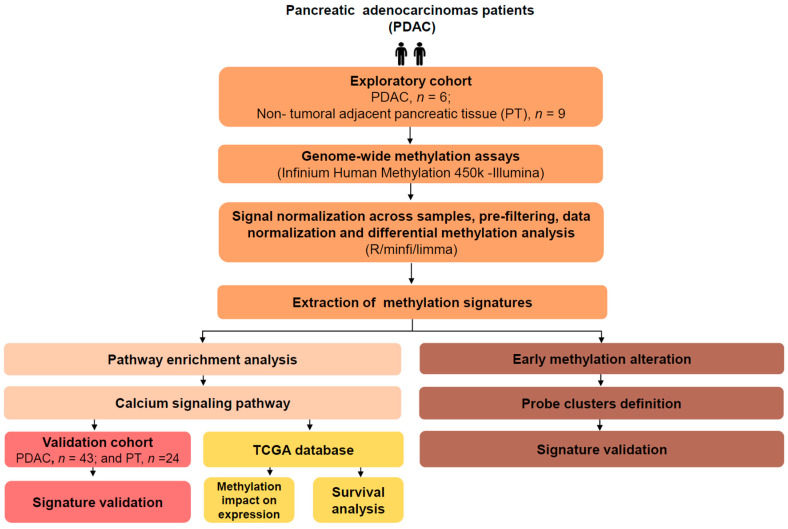
Flow chart illustrating the overall design of the study.

We performed a genome-wide DNA methylation profiling in an exploratory cohort using the 450 K BeadChip Array platform (Illumina Infinium Human Methylation, San Diego, CA, USA). Six PDAC and nine PT samples with content (cellularity) ≥70% were included. Differentially methylated genes were selected, and validation was performed by pyrosequencing in a biological and technical validation cohort comprising a different set including 43 PDAC and 24 PT samples. The overall experimental design is summarized in Scheme 1.

### 4.2. DNA Isolation and Bisulfite Conversion

The PureLink Genomic DNA Kit (Thermo Fisher Scientific) was used to isolate the DNA from fresh frozen tissues according to the manufacturer’s protocol and eluted DNA was quantified using Qubit V2.0 (Invitrogen, Carlsbad, USA). DNA from each sample was bisulfite converted using the EZ-DNA methylation kit (Zymo Research Corporation, California) according to the manufacturers’ protocol.

### 4.3. Human Methylation 450 K Array and Data Preprocessing

PDAC and PT tissues were used to establish an exploratory cohort. Illumina Infinium Human Methylation450 k (HM450 K) Bead-Chips (Illumina, San Diego, CA, USA) were used for investigating the genome-wide DNA methylation profile. Raw data were subjected to quality control, prefiltering, signal normalization across samples, and normalization using the funnorm function (R packages minfi) [87,88]. The Infinium data generated in this study were deposited in Gene Expression Omnibus (GEO) database and are available under accession number (GSE149250).

### 4.4. Differential Methylation Analysis

Differential methylation analysis was performed with *limma* R/Bioconductor package, using the M-values matrix as input [89]. Correction for multiple testing was conducted with the Benjamini–Hochberg false discovery rate (FDR) procedure. Probes were annotated for approved gene symbols using annotation files provided by the manufacturer, and all probes with ambiguous annotations were removed from further analyses. The identification of DMPs in PDAC and PT samples was performed as previously described [90]. The DMPs were identified by using a cut-off of FDR-corrected *p*-values < 0.01 and an absolute difference between the means of the β-values (∆β) ≥ 0.2. To verify the methylation patterns in cases and controls, a hierarchical clustering analysis was applied to the M-values from DMPs using the complete linkage method and Euclidean distance, as implemented by the *heatmap*.2 function from *gplots* R package.

### 4.5. Functional Enrichment Analysis

DMPs were subject to functional enrichment analysis using pathway annotations from the KEGG [91,92] and the *clusterProfiler* package [93] in the R/Bioconductor environment. For this purpose, the gene symbol annotation of hyper- and hypomethylated probes were analyzed separately. Only the KEGG pathways with a minimum size of 30 annotated genes were considered for further analyses. Statistical significance for the enrichment of the KEGG pathways was estimated with a hypergeometric test and adjusted to account for the multiple hypotheses testing using the FDR procedure. Pathways with a FDR-corrected *p*-value < 0.05 were highlighted as potentially enriched for hypo- or hypermethylated genes. Results were visualized using Cytoscape v3.4.0.

### 4.6. Technical and Biologic Validation

In order to biologically and technically validate the array data, we performed the pyrosequencing (PyroMark Q96 ID-Qiagen) of selected Ca^2+^ pathway genes in tissue samples with ≥ 20% cellularity. Genes were chosen according to the following selection criteria: only genes with DMP which were exclusively hypo- or hypermethylated; genes associated with a high |∆β|; and/or genes with previously described roles in carcinogenesis. After applying this filter, five genes were selected: *RYR3* (hypermethylated) and *CALM2*, *CAMK2A*, *ITPR2* and *EGFR* (all of them hypomethylated).

### 4.7. TCGA Analysis

RNA-seq and methylation microarray (HM450 K) data were downloaded using the GDC Data Transfer Tool. Only patients who had both methylation and expression data for all the target genes were included. The unsupervised hierarchical clustering (heatmap with dendrograms) and KEGG pathway figures were built with the Complex Heatmap v.2.0 and KEGG graph v.1.44.0 packages, respectively, and the correlations (FDR-adjusted *p*-value < 0.05) were performed with the psych v.1.8.12 package. The promoter category includes probes located in the genomic region TSS1500 and TSS200. All the PDAC samples with available mRNA data (*n* = 146) were used to analyze the mRNA expression prognostic values of selected Ca^2+^ pathway genes in PDCA samples. Only stage IV cases (*n* = 4) were excluded for being unresectable. The mRNA expression of selected genes was divided by tertiles and the lower and higher tertiles were used to classify cases into low and high expression, respectively. Genes for which more than one third of the cases had no detectable expression were analyzed by comparing the groups of patients with no detectable expression vs. patients with detectable expression. For the estimation of univariate survival, we used the Kaplan–Meier survival curve and statistical significance between the two groups was calculated by the log-rank test. Only genes with log-rank *p*-values < 0.05 remained in the model. Lymph node invasion and residual tumor status after surgical resection were selected for multivariate analysis due to their statistically significant association with OS in the univariate analysis. Cox regression using the stepwise forward method was applied. All survival analyses were performed using the package “Survival” for R.

## 5. Conclusions

In summary, our results show that DNA methylation alterations are involved in the deregulation of the Ca^2+^ signaling pathway genes. These alterations impact gene expression, the overall survival of patients, and are already seen in tumor-adjacent tissue. Future studies should be performed to validate our findings, but the data presented here indicate a significant role of epigenetic alterations in the Ca^2+^ signaling pathway in PDAC.

## Supplementary Materials

The following are available online at https://www.mdpi.com/2072-6694/12/7/1735/s1, Figure S1: Functional enrichment of differentially methylated genes in pancreatic adenocarcinoma, Figure S2: Validation of the genome-wide DNA methylation results with pyrosequencing, Figure S3: Non-tumoral pancreatic tissue -like (PT-like) analysis, Figure S4: Methylation levels of 24 Ca^2+^ signaling pathway genes in PT, PT-like and PDAC samples, Table S1: Enriched KEGG terms for hypermethylated genes, Table S2: Enriched KEGG terms for hypomethylated genes, Table S3: PDAC clinicopathological features of the validation cohort (*N* = 43), Table S4: Correlation between probe methylation and gene expression, Table S5: Evaluation of the impact of the expression of Ca^2+^ signaling genes on pancreatic ductal adenocarcinoma overall survival, Table S6: Analysis of early DNA methylation alterations, Table S7: Clinicopathological features of pancreatic ductal adenocarcinoma patients included in the exploratory and validation cohort.

## Abbreviations

*ADCY8*	Adenylate Cyclase 8
*ADRA1A*	Adrenoceptor Alpha 1A
*AGTR1*	Angiotensin II Receptor Type 1
ASICs	Acid-Sensing Ion Channels
Ca^2+^	Calcium
*CACNA1A*	Calcium Voltage-Gated Channel Subunit Alpha1 A
*CACNA1B*	Calcium Voltage-Gated Channel Subunit Alpha1 B
*CACNA1C*	Calcium Voltage-Gated Channel Subunit Alpha1 C
*CACNA1D*	Calcium Voltage-Gated Channel Subunit Alpha1 D
*CACNA1H*	Calcium Voltage-Gated Channel Subunit Alpha1 H
*CALM2*	Calmodulin 2
*CAMK2A*	Calcium/Calmodulin Dependent Protein Kinase II Alpha
*CAMKK1*	Calcium/Calmodulin Dependent Protein Kinase Kinase 1
*CASQ2*	Calsequestrin 2
*CDO1*	Cysteine Dioxygenase Type 1
CRAC	Calcium Release-Activated Calcium Channels
DMPs	Differentially Methylated Probes
*EGFR*	Epidermal Growth Factor Receptor
ER	Endoplasmic Reticulum
FAK	Focal Adhesion Kinase
FDR	False Discovery Rate
*FOXE1*	Forkhead Box E1
GEO	Gene Expression Omnibus
*GRIN2A*	Glutamate Ionotropic Receptor NMDA Type Subunit 2A
HM450K	Human Methylation 450k
HR	Hazard Ratio
*HRH1*	Histamine Receptor H1
IP_3_	Inositol-1,4,5-Trisphosphate
IP_3_Rs	Inositol 1,4,5-Trisphosphate Receptors
*ITPKB*	Inositol-Trisphosphate 3-Kinase B
*ITPR1*	Inositol 1,4,5-Trisphosphate Receptor Type 1
*ITPR2*	Inositol 1,4,5-Trisphosphate Receptor Type 2
KEGG	Kyoto Encyclopedia of Genes and Genomes
*KRAS*	KRAS Proto-Oncogene, GTPase
Mg^2+^	Magnesium
NHE1	Sodium/Proton Exchanger-1
*NPTX2*	Neuronal Pentraxin 2
*ORAI1*	ORAI Calcium Release-Activated Calcium Modulator 1
*ORAI2*	ORAI Calcium Release-Activated Calcium Modulator 2
OS	Overall Survival
*P2RX2*	Purinergic Receptor P2X 2
*PCDH10*	Protocadherin 10
PDAC	Pancreatic Ductal Adenocarcinoma
*PDE1C*	Phosphodiesterase 1C
*PENK*	Proenkephalin
*PLCB1*	Phospholipase C Beta 1
*PRKCB*	Protein Kinase C Beta
PT	Pancreatic Non-Tumoral Tissue
PT-LIKE	Pancreatic Non-Tumoral-Like Tissue
RSEM	RNA-Seq by Expectation-Maximization
*RYR2*	Ryanodine Receptor 2
*RYR3*	Ryanodine Receptor 3
RyRs	Ryanodine Receptors
*S1004A*	S100 Calcium Binding Protein A4
SERCA	Sarcoendoplasmic Reticulum Ca^2+^ ATPases
*SFRP1*	Secreted Frizzled Related Protein 1
*SFRP2*	Secreted Frizzled Related Protein 2
*SLC8A3*	Solute Carrier Family 8 Member A3
SOCE	Store Operated Calcium Entry
*STIM*	Stromal Interaction Molecule
*STIM*	Stromal Interaction Molecule 1
TCGA	Cancer Genome Atlas
*TFPI2*	Tissue Factor Pathway Inhibitor 2
TNM	TNM Classification of Malignant Tumors
TRPCs	Transient Receptor Potential Channels
TRPVs	Transient Receptor Potential Vanilloid Channels
TSGs	Tumor Suppressor Genes
UTR	Untranslated Region
