# Supplementary material for: Calcium Signaling Alterations Caused by Epigenetic Mechanisms in Pancreatic Cancer: From Early Markers to Prognostic Impact
Source: Cancers (Basel). 2020 Jun 30;12(7):1735. doi: 10.3390/cancers12071735 (PMC7407273; doi:10.3390/cancers12071735)
Supplement: Supplementary file 1 [file cancers-12-01735-s001.zip › cancers-804321-supp-final/cancers-804321-supp-final.docx]

Supplementary Materials: Calcium Signaling Alterations Caused by Epigenetic Mechanisms in Pancreatic Cancer: From Early Markers to Prognostic Impact

Cleandra Gregório, Sheila Coelho Soares-Lima, Bárbara Alemar, Mariana Recamonde-Mendoza, Diego Camuzi, Paulo Thiago de Souza-Santos, Raquel Rivero, Simone Machado, Alessandro Osvaldt, Patricia Ashton-Prolla and Luis Felipe Ribeiro Pinto


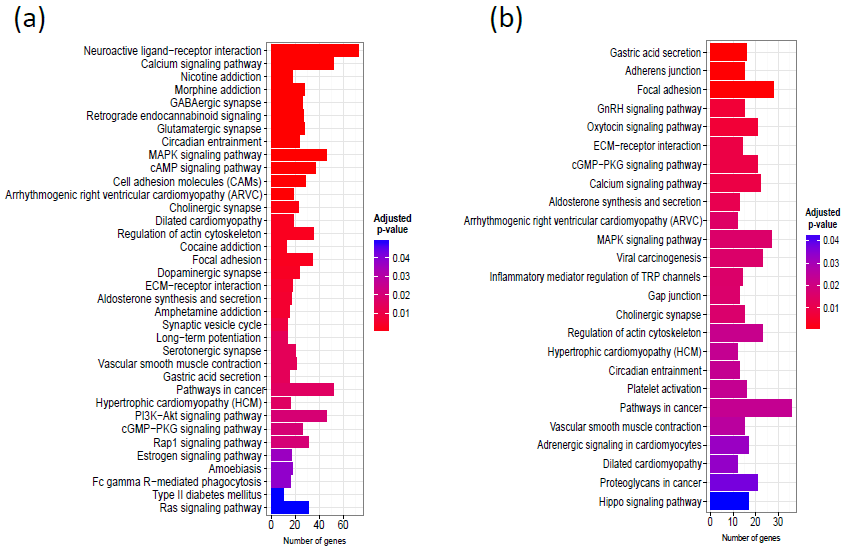


**Figure S1.** Functional enrichment of differentially methylated genes in pancreatic adenocarcinoma, (**a**) Pathways affected by gene hypermethylation (total of 611 functionally annotated genes); (**b**) Pathways affected by gene hypomethylation (total of 385 functionally annotated genes).

| 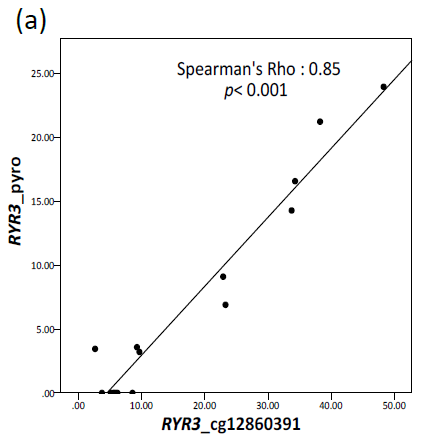 | 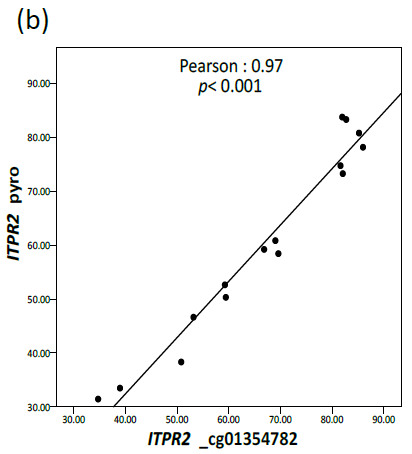 |
| --- | --- |
| 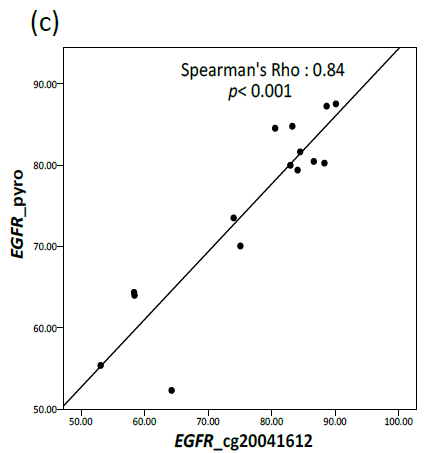 | 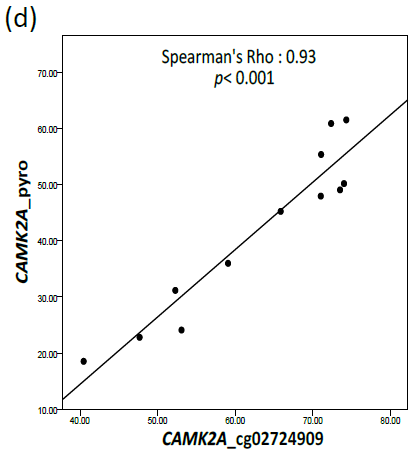 |
| 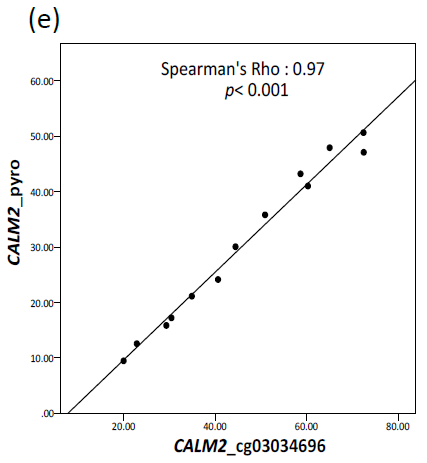 |  |

**Figure S2.** Validation of the genome-wide DNA methylation results with pyrosequencing. Plots display the correlation between the methylation level s o f each DMP determined by microarray (X-axis) and pyrosequencing (Y-axis) in the following genes: **(a):** *RYR3*; **(b):** *ITPR2*; **(c):** *EGFR*; **(d):** *CAMK2A*; **(e):** *CALM2*. The corresponding correlation coefficients as well as p-values are displayed in each plot.


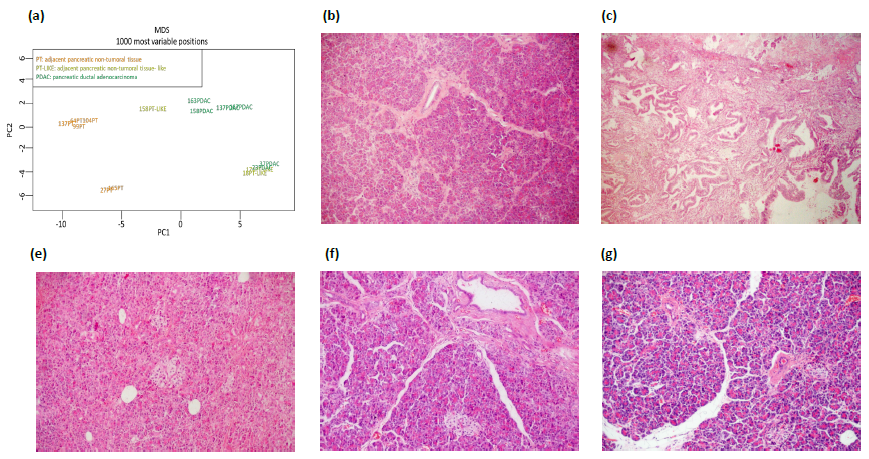


**Figure S3.** Non-tumoral pancreatic tissue -like (PT-like) analysis. Non-tumoral pancreatic tissue -like (PT-like) analysis. (**a**) Multi dimensional scaling (MDS) plot showing sample clustering. Hematoxylin and eosin staining (100x) of samples sections: (**b**) Representative slide of a non-tumoral pancreatic tissue (PT) used in this study. Pancreatic acini with preserved lobule architecture, interlobular septa, typical ducts and islets in between; (**c**) Representative slide of a pancreatic ductal adenocarcinoma (PDAC) used in this study. PDAC moderately differentiated, with irregularly shaped glands and isolated cells or in small groups, with an infiltrative pattern, stromal desmoplasia, and occasional mucin production; (**e**–**f**) PT samples 18, 158 and 171, respectively; with normal pancreatic morphology, likewise the (a) section. PC, principal component.

| 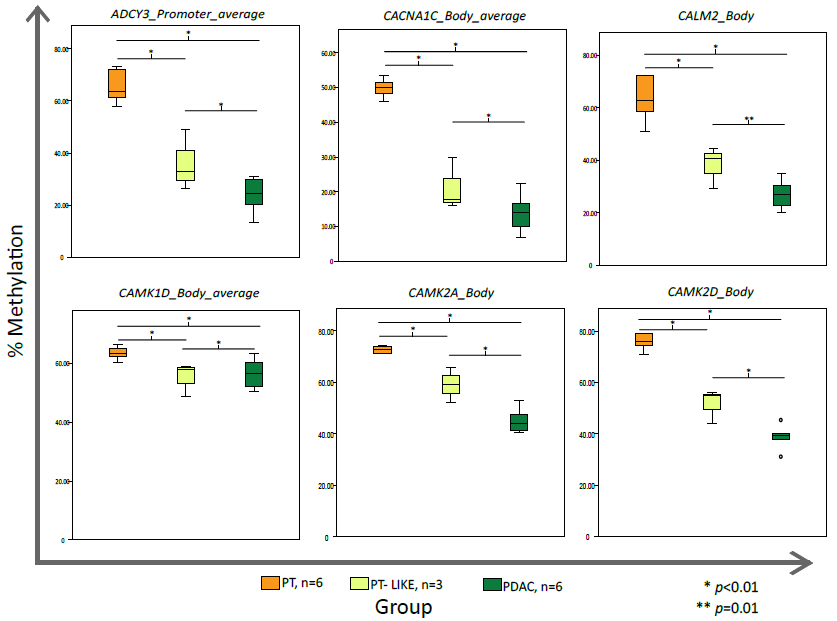 |
| --- |
| 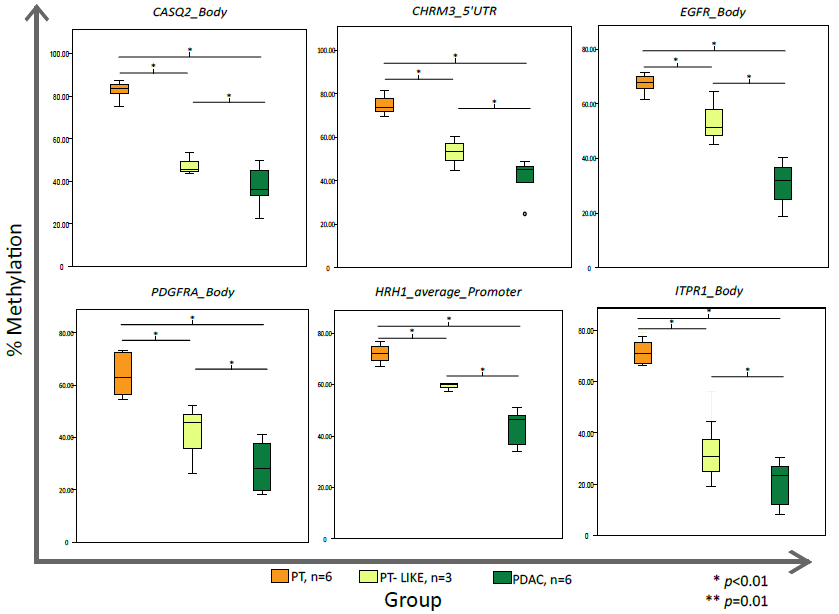 |
| 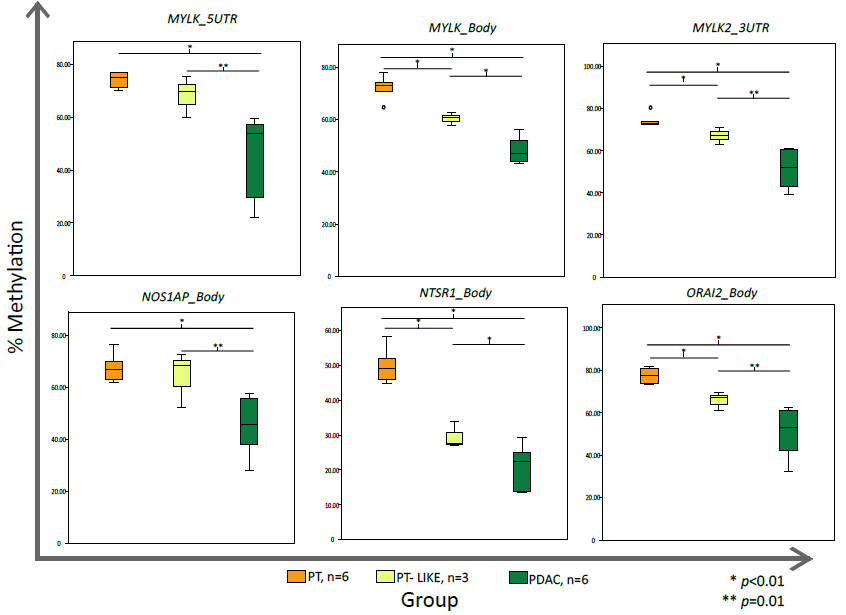 |
| 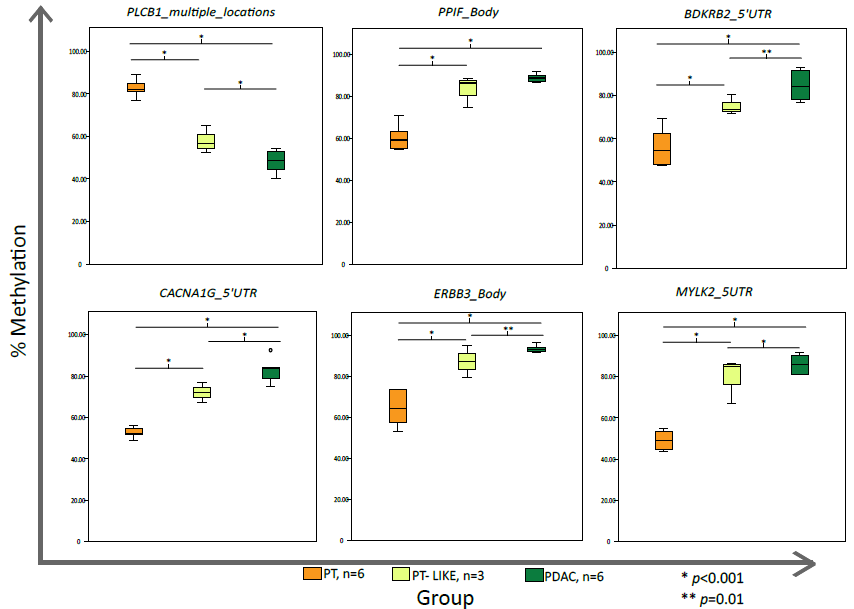 |
| 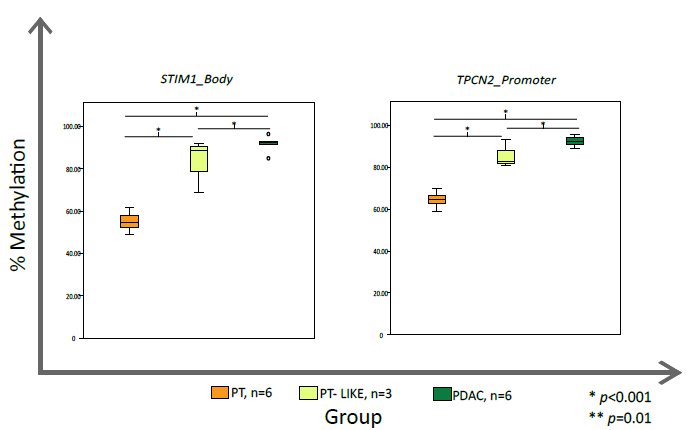 |

**Figure S4.** Methylation levels of 24 Ca^2+^ signaling pathway genes in PT, PT-like and PDAC samples. Boxplots represent the overall methylation percentage of PT, PT-like and PDAC of 26 differentially methylated probes of the Calcium signaling pathway from cluster analysis. Individual genes are depicted on top of each plot. *p*-value was calculated using the Generalized Estimating Equations.

**Table S1.** Enriched KEGG terms for hypermethylated genes.

**Table S2.** Enriched KEGG terms for hypomethylated genes.

**Table S3.** PDAC clinicopathological features of the validation cohort (*n* = 43).

**Table S4.** Correlation between probe methylation and gene expression.

**Table S5.** Evaluation of the impact of the expression of Ca^2+^ signaling genes on pancreatic ductal adenocarcinoma overall survival.

**Table S6.** Analysis of early DNA methylation alterations.

**Table S7.** Clinicopathological features of pancreatic ductal adenocarcinoma patients included in the exploratory and validation cohort.

| 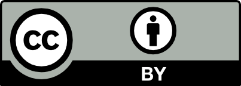 | © 2020 by the authors. Submitted for possible open access publication under the terms and conditions of the Creative Commons Attribution (CC BY) license (http://creativecommons.org/licenses/by/4.0/). |
| --- | --- |
